# Motivational Influences on Health, Well-Being, and Lifestyle: Validation of the Spanish Version of the Treatment Self-Regulation Questionnaire in Four Health Domains

**DOI:** 10.3390/ejihpe13110164

**Published:** 2023-10-26

**Authors:** Macarena De los Santos-Roig, Claudia Fusinato-Ponce, Manuel Fernández-Alcántara

**Affiliations:** 1 Department of Methodology of Behavioral Sciences, Faculty of Psychology, University of Granada, 18071 Granada, Spain; 2 Mind, Brain and Behaviour Research Centre, CIMCYC, University of Granada, 18071 Granada, Spain; 3 Hospital General Universitario Santiago Apóstol de Vitoria, 01004 Vitoria-Gasteiz, Spain; claudia.fusinatoponce@osakidetza.eus; 4 Department of Health Psychology, University of Alicante, 03080 Alicante, Spain; mfernandeza@ua.es

**Keywords:** autonomous motivation, scale development, validity, reliability, self-determination theory

## Abstract

Background: Motivation is a central concept in self-determination theory (SDT). The Treatment Self-Regulation Questionnaire (TSRQ), which assesses motivation (autonomous, controlled, etc.), has been widely used. However, less is known about its applicability to samples such as college students, who may be at risk of having unhealthy behavior in many areas (including smoking, poor dietary habits, alcohol, or tobacco consumption). As this population is transitioning to adulthood, research is needed to understand motivation and changing health patterns. In addition, the lack of instruments for this population in Spain has made the measurement validation process a priority. The purpose of this psychometric study was to adapt the TSRQ to Spanish college students and to examine its structural and validity across four health domains. Methods: Two samples of Spanish college students (*n* = 347 and *n* = 244) agreed to participate in the study. Participants completed a booklet containing measures of motivation, well-being, general health, anxiety, depression, and lifestyle. Results: CFA supported a five-dimensional structure in each domain. Reliability values were also adequate for each questionnaire. Regarding other sources of validity, statistically significant relationships between self-determination, health, and well-being were clearly confirmed, and autonomy was a significant predictor of lifestyle. Conclusions: The Spanish version of the TSRQ showed adequate psychometric properties (dimensionality and internal structure, reliability, and validity evidence regarding its relationships with other constructs) in college students. The Spanish TSRQ will provide future research aimed to understand the motivational role in college students’ health behavior and well-being.

## 1. Introduction

The World Health Organization’s (WHO) recommendations on the importance and influence of a healthy lifestyle in the prevention of chronic diseases are clear. In Western societies, however, one third of these health recommendations and suggestions are not followed or adhered to in the long term [1,2].

One of the theories used to explain the promotion of, and barriers to, healthy behaviors is Self-Determination Theory (SDT). In SDT, motivation is the central concept used to predict self-determined (or autonomous) behaviors [3]. In this context, autonomous motivation is defined as engaging in a particular behavior because it is perceived to be in line with one’s intrinsic goals. By contrast, controlled motivation refers to the pursuit of behaviors for external reasons, such as rewards, social approval, punishment, or feelings of guilt [4].

Autonomous motivation is positively correlated with well-being, physical health, and psychological functioning across a number of life domains [5,6,7] and negatively correlated with risky sexual behaviors [8]. As such, people who maintain their health for self-determined reasons (i.e., personal values) enjoy better health and well-being and are less exposed to risks. Furthermore, higher autonomous motivation has shown moderate positive correlations with physical activity and dietary intake (i.e., fruit and vegetables), whereas controlled forms of motivation have shown weak negative correlations with these variables [9,10]. Measuring this in young people is therefore important [11,12], but especially so in college students, who nowadays have their learning influenced by social media and digital manipulation and for whom likely only intrinsic values and critical thinking skills could help in making health decisions autonomously [13]. Indeed, when young people start studying at university, they often have to move to another city, away from parental control, and start making decisions and behaving according to their own rules. As pointed out by Graham et al. [14], it is during this transition from adolescence to adulthood that poor eating habits, for example, can develop. Despite some studies [15], little is known about the factors that influence the development of healthy or unhealthy behaviors at this stage of life. There is, therefore, a need for research in this population.

The Treatment Self-Regulation Questionnaire, TSRQ [16,17], was developed to assess autonomous and controlled motivation. Levesque et al. [18] analyzed the structure of the 15-item version in relation to smoking cessation, dietary improvement, and physical exercise. They found that the “identification” and “integration” dimensions tended to cluster well together as “autonomous motivation”, while “introjections” and “external” (both part of what is theoretically called “controlled motivation”) tended to separate. Using this structure, they confirmed a four-dimensional model that included “amotivation” and remained stable across all health domains. Similar results were obtained in Spain, where Férriz et al. [11] used the TSRQ to assess healthy lifestyles in adolescents. However, there is still uncertainty about how the scale works in college student populations and what the potential differences are when assessing different health behaviors. Exploring these issues would provide evidence of the generalizability of the scale structure [4].

For these reasons, the aim of the present psychometric study was to validate the use of the Spanish version of the TSRQ in college students and to (i) confirm its four-factor structure, (ii) test its reliability, and (iii) confirm its relationships with several measures of health, well-being, and lifestyle as in previous research. The TSRQ assessed motivation in four health domains (i.e., smoking, diet, exercise, and alcohol), and it is expected that evidence will be found in all four. Taking into account previous studies and evidence of structural validity [11,18,19], our hypothesis was that (H1) the TSRQ will have a four-factor structure: autonomous motivation (clustering the identification and integration dimensions), introjected motivation, external motivation, and amotivation. In addition, given the validity and previous research on autonomous motivation and health behavior, our hypothesis was that (H2) high scores in self-determination (or later, autonomy as a composite index of autonomous and controlled motivation) will be correlated with better physical health, lower psychological distress (i.e., anxiety and depression), and therefore greater overall well-being. Finally, in light of previous research on the relationship between self-determination and lifestyle, we expected that (H3) high scores in autonomy will be predictive of a healthy lifestyle.

## 2. Materials and Methods

### 2.1. Samples

Two different samples were recruited for the study (*n* = 591). Inclusion criteria for participants were to be a college student, to speak Spanish as a mother language and to not suffer from any sensorial or intellectual disability. Sample 1 (construction study and external validity sample, S1) consisted of 347 college students, the majority of whom were women (*n* = 275, 79.3%), with a mean age of 20.35 years (SD = 3.51). Sample 2 (replication study and external validity sample, S2) comprised 244 college students, the majority of whom were women (*n* = 203, 83.2%), with a mean age of 20.75 years (SD = 4.64). Considering the standards of factorial analysis, which suggest a minimum sample size of 10 participants per item, each sample should at least have 130 participants [20].

### 2.2. Instruments

#### 2.2.1. Spanish Version of the Treatment Self-Regulation Questionnaire, TSRQ [17]

The back-translated version contains 15 items describing motivations or reasons (autonomous and controlled) for staying healthy in four domains: smoking, diet, exercise and alcohol. Every item refers to a different motivation that must be rated on a Likert scale from 1 (“totally false”) to 7 (“completely true”). The instructions vary depending on the specific behavior being assessed. The original version has acceptable internal consistency, *α* = 0.73 [21]. All participants completed this measure. The alpha indexes obtained in our study are presented later.

#### 2.2.2. Ryff Scales of Psychological Well-Being [22]

This is a 29-item instrument assessing six aspects of perceived well-being: (a) Self-Acceptance, (b) Positive Relations, (c) Autonomy, (d) Environmental Mastery, (e) Purpose in Life, and (f) Personal Growth. Scores range from 1 (“strongly disagree”) to 6 (“strongly agree”). It was administered to S1, and most scales had adequate reliability indexes (with alphas ranging from 0.58 to 0.82), similar to those of the Spanish version [23].

#### 2.2.3. Scale of Psychological Well-Being, EBP [24]

The EBP consists of 65 items rated on a scale from 1 (“never”) to 5 (“always”). We used the overall psychological well-being subscale (30 items), which distinguishes between (a) life satisfaction and (b) positive–negative affect. The latter includes the Happiness, Hope, Health and Sociability subscales. In our study, it was administered to S1 and showed acceptable reliability (alpha ranged from 0.64 to 0.86). The original scale had high internal consistency (*α* = 0.93).

#### 2.2.4. General Health Questionnaire, SF-12 [25]

This is an abbreviated version of the SF-36 providing a subjective measure of physical and mental health. It is a 12-item scale assessing eight dimensions: (a) Physical Function, (b) Social Function, (c) Physical Role, (d) Emotional Role, (e) Mental Health, (f) Vitality, (g) Bodily Pain, and (h) General Health, plus two general components labeled Overall Physical Health and Overall Mental Health. High scores in the dimensions are an indication of better health. The Spanish adaptation [26] has shown internal consistency values greater than 0.70 and significant correlations between both versions of the scale [27]. In our study, it was administered to both S1 and S2 and showed low internal consistency (around 0.60 in both of the overall components).

#### 2.2.5. Hospital Anxiety and Depression Scale, HADS [28]

The Spanish version of the HADS [29] has two subscales (i.e., Anxiety and Depression), each consisting of seven items. High scores indicate the presence of more symptoms of anxiety and depression. It was administered to S2 and showed adequate internal consistency with values of 0.78 and 0.70 for each subscale, respectively.

#### 2.2.6. Lifestyle Assessment Scale, EEV [30]

The EEV was administered to S1 and assesses the frequency with which individuals engage in healthy behaviors. It is made up of 68 items rated on a four-point Likert scale. High scores in the dimensions indicate a healthier lifestyle. This instrument has reliability indexes ranging from *α* = 0.40 (for the management of free time subscale) to *α* = 0.77 (drug use). The subscales of interest for this study were (1) condition, physical activity, and sports; (2) eating habits; and (3) use of alcohol, tobacco, and other drugs, with reliability indexes of *α* = 0.60, *α* = 0.70, *α* = 0.70, and *α* = 0.77, respectively.

### 2.3. Procedure

Two bilingual specialists translated the original TSRQ scales (TSRQ—Smoking/Diet/Exercise/Alcohol) into Spanish, and then another two bilingual specialists translated them back into English. The English versions of the four scales were compared, and no differences were found between the original and back-translated versions [31]. The final versions and the other instruments were presented to participants in counterbalanced order. 

Data collection took place in college classrooms during school hours and with the participants’ consent. Data were collected at two points during the school year, in December and April. All those taking part earned additional marks for the subject. All participants filled out a pencil–paper-format booklet of questionnaires provided by the evaluators. The data collection process was single-blinded and controlled by constancy. So, participants did not know the real purpose of the study, and the procedure and conditions (place, time, instructions, evaluators, etc.) for administering the tests were identical for both samples. The retest evaluation (conducted with S2 only) took place one month after the test using only the TSRQ scales.

For research reasons beyond the scope of this paper, the following instruments were applied to each of the samples: TSRQ, RYFF, EBP, EEV, and SF-12 to S1 and TSRQ, RYFF, EBP, SF-12, and HADS to S2.

This research was approved by the University of Granada´s Research Ethics Committee (620/CEIH/2018).

### 2.4. Data Analysis

To verify the structure of the TSRQ, we performed confirmatory factor analyses with S1 and S2 in each of the four health domains. The overall fit of the model was assessed using the joint criteria proposed by Hu and Bentler [32]: Comparative Fit Index (CFI) ≥ 0.95, Root Mean Square Error of Approximation (RMSEA) ≤ 0.06, and Standardized Root Mean Square (SRMR) ≤ 0.08. However, other authors have suggested that CFI values close to 0.90 are indicative of a model with a good fit [33]. Given the general non-normality of item responses, we used a robust estimation method (i.e., MLR).

Pearson correlations were performed to provide evidence of the reliability and validity of the TSRQ scores: (1) the stability of the scores using the test–retest method and (2) the validity of the TSRQ scores by confirming the relationships between self-determined motivation (via the Autonomy Index [7,34]) and both the health and well-being variables. The third objective was to test the predictive effect of autonomy on healthy lifestyles. We therefore carried out multiple linear regression analyses. SPSS V.23 and MPLUS statistical software were used [35]. The significance level was set at *p* < 0.05.

## 3. Results

### 3.1. Factor Structure of the TSRQ: Using the Construction Sample (S1) and the Replication Sample (S2)

Several CFAs were conducted to explore the proposed (four-dimensional) structure and to compare the goodness-of-fit between this and two other models (a three-dimensional model and a five-dimensional model, respectively) in S1. The process was then repeated with the replication sample (S2).

To guard against confirmation bias [36], the alternative models were compared with the model under evaluation using the Akaike Information Criterion (AIC): (1) Model 1, based on three overall theoretical components (“autonomous motivation”, “controlled motivation”, and “amotivation”); (2) Model 2, based on four sub-dimensions confirmed by previous research [11,17,18] (“integrated” and “identified” regulation—clustered as “autonomous motivation”—“introjection”, “external”, and “amotivation”); and (3) Model 3, based on the five sub-dimensions of the scale derived originally from SDT (“integrated”, “identified”, “introjection”, “external” motivation, and “amotivation”). In all these models, the item “Because it is easier to do what I am told than think about it” (amotivation) was included as part of external regulation because of both its semantic content and its low item–total correlation with the original dimension. This is also in line with previous research [11,18,19].

**Figure 1:**
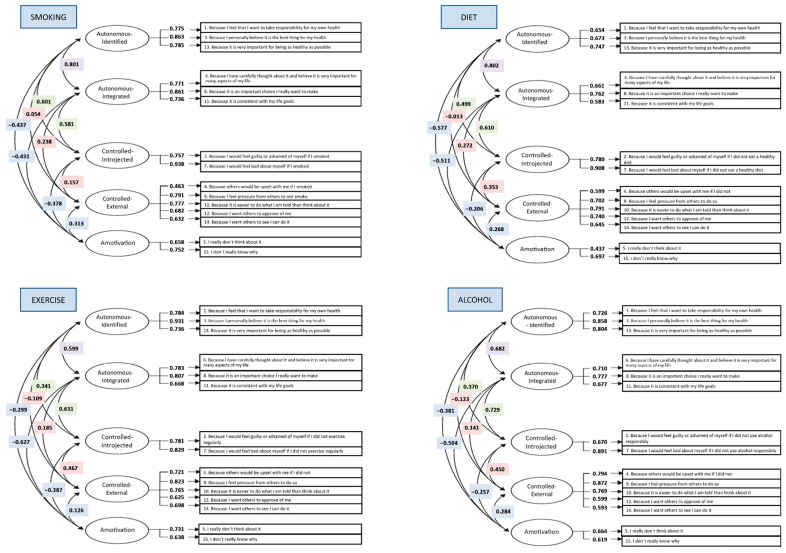
Regression coefficients for the CFA in Model 3 for S1.

**Figure 2:**
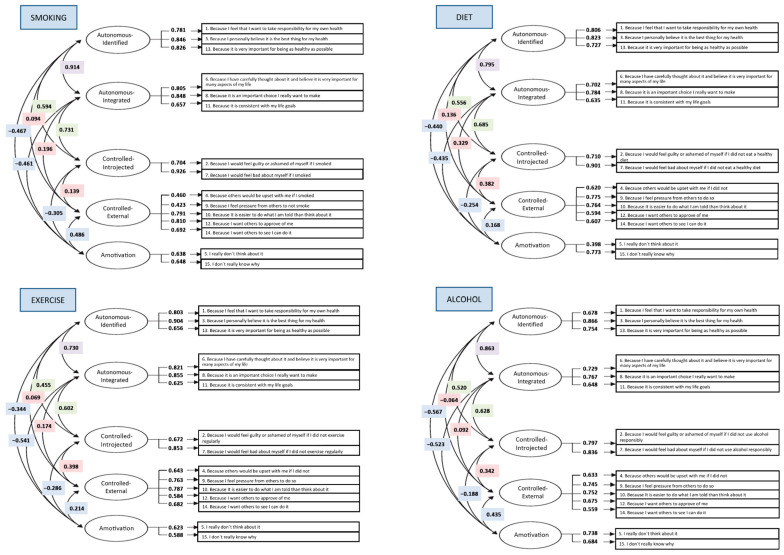
Regression coefficients for the CFA in Model 3 for S2.

Table 1 shows the goodness-of-fit indexes of each model. Models 1 and 2 showed a poor fit in both samples. Model 3 obtained better fit indexes in S1 and S2, with a CFI ranging from 0.91 to 0.95. Error values for the TSRQ in the four health domains were also generally adequate (RMSEA: 0.050–0.074 and SRMR: 0.055–0.063). The five-dimensional model (Model 3) also appeared to be the best fit when taking into account AIC. Factor loadings were significant (*p* < 0.001). Regression coefficients ranged from 0.40 to 0.94 (see Figure 1 and Figure 2). The results obtained for Model 3 were also adequate (*Χ^2^*/gl < 5, [37]) in the four health domains.

### 3.2. Reliability Indexes of the Resulting Model

Cronbach’s α indexes were high for nearly all five dimensions, with values ranging from 0.70 to 0.85 in all TSRQ domains. The “amotivation” dimension had the lowest alpha values (*α* = 0.46 to *α* = 0.63).

Test–retest correlations ranged from *r* = 0.55 to *r* = 0.71 for “integration”, “identification”, “introjection”, and “external” in the four TSRQ domain scales. The “amotivation” scale showed a test–retest correlation ranging from *r* = 0.42 to *r* = 0.55.

### 3.3. Correlations between Self-Determination (Autonomy Index) and Well-Being, Perceived Health, and Mental Health

To test the validity hypothesis with a more manageable self-determination score, TSRQ items scores were combined into a single relative Autonomy Index (also called the Self-Determination Index) by using the formula 2 × integration + identification - introjection − 2 × external [7,34]. Amotivation was not included.

With respect to perceived health (SF-12), the results show a small but positive and significant correlation (*r* = 0.10, *p* < 0.01) between physical health and autonomy in some TSRQ scales (see Table 2). Overall mental health was significantly correlated with self-determination in most health domains (*r* of around 0.10, *p* < 0.05). Significant (*p* < 0.05) and positive correlations were also found between autonomy and the two well-being scales (i.e., EBP and Ryff), with values ranging from *r* = 0.20 to *r* = 0.30. Similar results were obtained for anxiety and depression (HADS) where negative correlations were around −0.15 for anxiety and around −0.17 for depression (*p* < 0.05).

### 3.4. Self-Determination as a Predictor of a Healthy Lifestyle

As shown in Table 3, all four TSRQ domains were significantly correlated with a healthy lifestyle, with the exception of condition, physical activity, and sports. Multiple linear regressions also showed that the Autonomy Index was a significant predictor of some dimensions of a healthy lifestyle. On the one hand, having more autonomous reasons to quit smoking (TSRQ—Tobacco), to adopt a healthier diet (TSRQ—Diet), and to moderate alcohol consumption (TSRQ—Alcohol) were predictors of better eating habits (*β*_TSRQ-Smoking_ = 0.15, *t* = 2.19, *p* < 0.05, β_TSRQ-Diet_ = 0.18, t = 2.25, *p* < 0.05, and *β*_TSRQ-Alcohol_ = 0.20, *t* = 3.04, *p* < 0.01, respectively). On the other hand, having autonomous reasons to moderate alcohol consumption (TSRQ—Alcohol) was also predictive of healthy behaviors related to smoking/alcohol consumption and the use of other drugs, such as coffee, narcotics, and so on (*β*_TSRQ-Alcohol_ = 0.28, *t* = 4.09, *p* < 0.01). However, none of the self-determined reasons for healthy behaviors (TSRQ—all domains) were significant predictors of condition, physical activity, and sports. 

## 4. Discussion

The purpose of this psychometric study was to adapt the TSRQ to Spanish college students and to examine its validity across four health domains. To our knowledge, this is one of the first studies to focus on confirming the structure of the TSRQ in four health domains among college students.

The analyses performed confirmed that the TSRQ had adequate psychometric properties, in line with previous studies [11,18]. In terms of the structure of the TSRQ, confirmatory factor analysis showed that the best fit was the five-dimensional model derived from SDT by Ryan and Deci [38]. In this model, the identification and integration forms of extrinsic motivation were included as separate dimensions (and not grouped together as autonomous motivation). Contrary to our hypothesis, the four-dimensional model was not confirmed, and item 10 (“Because it is easier to do what I am told than think about it”) was even relocated to the external regulation subscale, as found by other authors [11,19]. Neither the three-dimensional nor the four-dimensional model achieved adequate fit indexes in our study. Indeed, the model with the best fit (i.e., the five-dimensional one) was confirmed both in the four health domains (smoking, diet, exercise, and alcohol consumption) and in the two independent samples used (S1 and S2). Although some studies of the TSRQ have found that the four-factor model has a better fit [11,18], the reality is that others have also found poor fit indexes when confirming this model [19,39].

### 4.1. Relationships between Self-Determination, Health, and Well-Being

According to SDT, people who are autonomously regulated exhibit higher levels of well-being, mental health (i.e., lower levels of depression and anxiety and better quality of life), better physical health, and healthier behaviors [38]. The present study’s results are in line with the research on self-determination and the various components of physical health [40,41], overall well-being [42,43], and the adoption or continuation of healthy behaviors [44,45]. The correlations in these studies are similar, also in terms of magnitude, to our main findings.

A recent meta-analysis carried out by Sheeran et al. [46] tested the moderating role of autonomous motivation in behavioral change. Significant effects were found for physical activity, sedentary behavior, diet, alcohol consumption, and smoking cessation using SDT-based interventions. We also used the HADS scale and found that people with lower levels of autonomy had higher rates of anxiety and depression. Other authors have reported similar associations with mental health [6,47]. In summary, our results are consistent with all these findings and show the good performance of the TSRQ in all four health domains.

### 4.2. Self-Determination and a Healthy Lifestyle

Regression analyses showed that self-determination was a predictor of healthy behaviors in two of the three dimensions under consideration (eating habits and drug use). The results relating to eating habits are particularly noteworthy: people with greater autonomy over diet, smoking, and alcohol consumption had better eating habits. In terms of diet, these findings are consistent with those of a recent meta-analysis on college students, which found the university environment appears to make healthy eating more challenging. As found by Maillet and Grouzet [15], students’ satisfaction of psychological needs may explain observed changes in self-regulation, motivation, and eating habits. This means that during this transition period, students tend to eat less food, less healthily, and less regularly. These changes occur mainly among students who move into university accommodation or off-campus housing, with little change in dietary behavior among students who continue to live at home. However, incoming students with more autonomous behavior (due to food literacy and/or experience of independent living) appear to be less likely to experience these changes. Autonomous motivation was also found to be a significant predictor of tobacco and alcohol use. These results confirm previous findings. In particular, Jerković et al. [48] showed that participants with higher autonomous motivation and certain personality traits (such as conscientiousness) were less likely to use cannabis. On the other hand, Richards et al. [49] found that students with more self-determined motivations to drink responsibly had a higher likelihood of using alcohol protective behavioral strategies in their day-to-day lives.

Contrary to expectations, exercising on a daily basis was not predicted by any of the autonomous reasons included in the TSRQ’s four health domains (nor by the TSRQ—Exercise or any other TSRQ scale). This is not consistent with previous research [45]. Part of the explanation for this may lie in the measurement instrument used in our study (the EEV): the exercise dimension is made up of mixed items relating to exercise, weight control, and rest.

The present study has important implications. The TSRQ can be highly valuable when applied to young adults to assess their healthy behaviors. This will enable the development of prevention and intervention strategies in line with what occurs in adolescents [11]. Also, future research should be conducted to explore the mediational role of other variables that impact health behaviors and motivation in college students. For example, the type of achievement goals has been related to intrinsic and extrinsic motivation with contradictory results [50]. Furthermore, the influence of social media and digital manipulation on the process of making health decisions should be explored [13]. The TSRQ may be an appropriate instrument to test further hypotheses in young adults.

Finally, the cross-sectional and associative design of the present study limits the explanatory power and generalizability of the results. Future studies of a more experimental nature or with more complex statistical analyses (e.g., mediation or moderated mediation analyses) would help to provide more precise explanations relevant to determining the role of autonomy in the acquisition of healthy behaviors. Moreover, the reliability values in some of the subscales used in the present research were relatively low, with values less than 0.70. In addition, while the sample was made up of college students, the majority were women, so further research is needed in order to generalize the results. Also, future studies are needed considering the level of psychopathology and how these scales respond in the clinical population or in college students with learning disabilities.

In conclusion, the Spanish adaptation of the TSRQ scales has provided more than sufficient evidence to justify its use in the study of motivation for the acquisition and maintenance of healthy behaviors in college students.

## Figures and Tables

**Table 1:** Confirmatory factor analysis of the TSRQ health domains conducted with S1 (*n* = 347) and S2 (*n* = 244).

		**Smoking**					**Diet**				
**Model**		***Χ^2^*** **/df**	**CFI/TLI**	**RMSEA (90% CI)**	**SRMR**	**AIC**	***Χ^2^*** **/df**	**CFI/TLI**	**RMSEA (90% CI)**	**SRMR**	**AIC**
**1**	S1	6.71	0.706/0.645	0.129 [0.119–0.139]	0.150	18,885.86	5.60	0.698/0.635	0.116 [0.106–0.126]	0.120	19,084.03
	S2	3.81	0.735/0.680	0.107 [0.905–0.120]	0.150	13,559.76	3.84	0.762/0.713	0.109 [0.096–0.121]	0.114	13,302.58
**2**	S1	3.34	0.833/0.854	0.083 [0.072–0.094]	0.070	18,530.30	2.79	0.886/0.858	0.072 [0.061–0.083]	0.069	18,794.43
	S2	1.79	0.928/0.910	0.057 [0.042–0.072]	0.070	13,339.63	2.20	0.903/0.878	0.071 [0.057–0.085]	0.068	13,145.98
**3**	**S1**	**2.51**	**0.928/0.906**	**0.067 [0.055–0.078]**	**0.063**	**18,483.30**	**2.48**	**0.910/0.882**	**0.066 [0.054–0.077]**	**0.059**	**18,753.22**
	**S2**	**1.61**	**0.947/0.930**	**0.050 [0.033–0.066]**	**0.068**	**13,317.40**	**1.80**	**0.936/0.916**	**0.059 [0.043–0.074]**	**0.060**	**13,106.44**
		**Exercise**					**Alcohol**				
**Model**		***Χ^2^*** **/df**	**CFI/TLI**	**RMSEA (90% CI)**	**SRMR**	**AIC**	***Χ^2^*** **/df**	**CFI/TLI**	**RMSEA (90% CI)**	**SRMR**	**AIC**
**1**	S1	7.50	0.673/0.605	0.138 [0.128–0.148]	0.134	17,923.35	6.20	0.717/0.658	0.123 [0.113–0.133]	0.129	17,166.90
	S2	3.96	0.755/0.704	0.110 [0.098–0.123]	0.110	12,445.30	3.72	0.743/0.690	0.106 [0.904–0.118]	0.132	11,880.40
**2**	S1	5.55	0.779/0.723	0.115 [0.105–0.126]	0.088	17,714.85	4.25	0.829/0.786	0.097 [0.087–0.108]	0.079	16,960.36
	S2	2.63	0.870/0.837	0.082 [0.069–0.095]	0.057	12,315.73	2.07	0.902/0.878	0.066 [0.052–0.080]	0.069	11,693.81
**3**	**S1**	**2.89**	**0.913/0.885**	**0.074 [0.063–0.086]**	**0.061**	**17,478.74**	**2.43**	**0.928/0.906**	**0.065 [0.053–0.076]**	**0.055**	**16,834.76**
	**S2**	**1.61**	**0.953/0.938**	**0.050 [0.034–0.066]**	**0.049**	**12,225.03**	**1.96**	**0.916/0.890**	**0.063 [0.048–0.077]**	**0.064**	**11,676.33**

Note. TSRQ = Treatment Self-Regulation Questionnaire; *χ*^2^/gf = Wheaton et al.´s (1977) chi-square, CFI = Comparative Fit Index; TLI = Tucker-Lewis Index; RSMSEA = Root Mean Square Error of Approximation; CI = Confidence Interval; SRMR = Standardized Root Mean Square residual; AIC = Akaike Information Criterion.

**Table 2:** Means, Standard Deviations, and Inter-Correlations between TSRQ scales, perceived health, and well-being scales.

	Mean	SD	(1)	(2)	(3)	(4)
Autonomy Index (*n* = 591)						
(1)TSRQ—Smoking	35.85	16.72				
(2) TSRQ—Diet	39.27	17.03	0.60 **			
(3) TSRQ—Exercise	41.34	16.05	0.56 **	0.75 **		
(4) TSRQ—Alcohol	31.37	18.55	0.57 **	0.53 **	0.53 **	
Perceived Health, SF-12 (*n* = 591)						
Physical Function	93.29	15.34	0.06	0.02	0.04	0.07
Physical Role	82.74	32.25	0.06	0.08 *	0.12 **	0.06
Bodily Pain	89.41	17.84	0.14 **	0.06	0.08 *	0.04
General Health	70.55	19.06	0.17 **	0.08 *	0.11 **	0.15 **
Vitality	58.51	21.54	0.13 **	0.08 *	0.14 **	0.11 **
Social Function	79.78	22.88	0.11 **	0.10 *	0.10 *	0.08 *
Emotional Role	56.00	44.26	0.13 **	0.10 *	0.10 *	0.13 **
Mental Health	54.85	10.41	−0.00	−0.00	−0.02	−0.03
SF-12 Total Physical Health	57.49	7.35	0.10 *	0.05	0.10 *	0.07
SF-12 Total Mental Health	36.07	10.10	0.12 **	0.09 *	0.09 *	0.10 *
Psychological Well-Being						
EBP (*n* = 346)						
Happiness	21.32	4.52	0.14 **	0.15 **	0.18 **	0.17 **
Hope	16.12	4.12	0.14 **	0.07	0.13 *	0.15 **
Health	14.22	3.10	0.18 **	0.12 *	0.12 *	0.15 **
Sociability	16.59	2.43	0.13 *	0.16 **	0.19 **	0.13 *
Life Satisfaction	42.07	7.10	0.23 **	0.18 **	0.19 **	0.20 **
Total EPB	110.35	18.00	0.21 **	0.17 **	0.20 **	0.20 **
RYFF (*n* = 346)						
Self-Acceptance	17.68	18.84	0.22 **	0.23 **	0.24 **	0.18 **
Positive Relations	32.26	3.92	0.17 **	0.22 **	0.26 **	0.15 **
Autonomy	25.47	5.37	0.29 **	0.25 **	0.28 **	0.16 **
Environmental Mastery	16.91	5.51	0.26 **	0.22 **	0.25 **	0.26 **
Personal Growth	22.35	3.10	0.33 **	0.35 **	0.33 **	0.29 **
Purpose in life	19.70	3.37	0.25 **	0.25 **	0.24 **	0.20 **
Total Ryff	125.46	4.66	0.34 **	0.34 **	0.36 **	0.26 **
HADS (*n* = 241)						
Anxiety	7.34	3.68	−0.17 **	−0.15 **	−0.18 **	−0.12
Depression	2.96	2.60	−0.21 **	−0.15	−0.21 **	−0.10

Note. SD = Standard Deviation, SF-12 = General Health Questionnaire SF-12, EBP = Perceived Well-Being Questionnaire, Ryff = Ryff Well-Being Scales, HADS = Hospital Anxiety and Depression Scale, (*) *p* < 0.05, (**) *p* < 0.01.

**Table 3:** Means, Standard Deviations, and Inter-Correlations between all components of the EEV (Lifestyle Assessment Scale) and TSRQ scales (*n* = 337).

			TSRQ Autonomy Index			
EEV Healthy Lifestyle	Mean	SD	Smoking	Diet	Exercise	Alcohol Use
Condition, Physical Activity, and Sports	16.94	3.32	0.010	0.040	0.080	0.046
Free time	10.26	2.10	0.125 *	0.178 **	0.170 **	0.131 **
Care	45.26	8.12	0.209 **	0.137 **	0.103 *	0.202 **
Eating habits	35.72	6.09	0.331 **	0.300 **	0.218 **	0.336 **
Drug use	17.82	5.12	0.233 **	0.115 *	0.158 **	0.312 **
Sleeping habits	21.43	4.04	0.164 **	0.057	0.081	0.132 **
TOTAL	147.55	17.46	0.341 **	0.246 **	0.227 **	0.359 **

Note. SD = Standard Deviation, (*) *p* < 0.05, (**) *p* < 0.01.

## Data Availability

Data are available under request to authors.
